# Effect of selective β-adrenoceptor blockade and surgical resection of the celiac-superior mesenteric ganglion complex on delayed liquid gastric emptying induced by dipyrone, 4-aminoantipyrine, and antipyrine in rats

**DOI:** 10.1590/1414-431X20155011

**Published:** 2016-02-02

**Authors:** A.M. Vinagre, E.F. Collares

**Affiliations:** 1 Núcleo de Medicina e Cirurgia Experimental, Faculdade de Ciências Médicas, Universidade Estadual de Campinas, Campinas, SP, Brasil; 2 Departamento de Pediatria, Faculdade de Ciências Médicas, Universidade Estadual de Campinas, Campinas, SP, Brasil

**Keywords:** Gastric emptying, Dipyrone, 4-aminoantipyrine, Antipyrine, β_2_-Adrenoceptor, Prevertebral sympathetic ganglia

## Abstract

There is evidence for participation of peripheral β-adrenoceptors in delayed liquid gastric emptying (GE) induced in rats by dipyrone (Dp), 4-aminoantipyrine (AA), and antipyrine (At). The present study aimed to determine whether β-adrenoceptors are involved in delayed GE induced by phenylpyrazole derivatives and the role of the prevertebral sympathetic nervous system in this condition. Male Wistar rats weighing 220-280 g were used in the study. In the first experiment rats were intravenously pretreated with vehicle (V), atenolol 30 mg/kg (ATE, β_1_-adrenergic antagonist), or butoxamine 25 mg/kg (BUT, β_2_-adrenergic antagonist). In the second experiment, rats were pretreated with V or SR59230A 2 mg/kg (SRA, β_3_-adrenergic antagonist). In the third experiment, rats were subjected to surgical resection of the celiac-superior mesenteric ganglion complex or to sham surgery. The groups were intravenously treated with saline (S), 240 µmol/kg Dp, AA, or At, 15 min after pretreatment with the antagonists or V and nine days after surgery. GE was determined 10 min later by measuring the percentage of gastric retention (%GR) of saline labeled with phenol red 10 min after gavage. The %GR (means±SE, n=6) values indicated that BUT abolished the effect of Dp (BUT+Dp *vs* V+Dp: 35.0%±5.1% *vs* 56.4%±2.7%) and At (BUT+At *vs* V+At: 33.5%±4.7% *vs* 52.9%±2.6%) on GE, and significantly reduced (P<0.05) the effect of AA (BUT+AA *vs* V+AA: 48.0%±5.0% *vs* 65.2%±3.8%). ATE, SRA, and sympathectomy did not modify the effects of treatments. These results suggest that β_2_-adrenoceptor activation occurred in delayed liquid gastric emptying induced by the phenylpyrazole derivatives dipyrone, 4-aminoantipyrine, and antipyrine. Additionally, the released neurotransmitter did not originate in the celiac-superior mesenteric ganglion complex.

## Introduction

The phenylpyrazole derivatives dipyrone, 4-aminoantipyrine, and antipyrine delay liquid gastric emptying (GE) in rats (1 2 3). The mechanisms involved in this process have not been clarified and appear to be complex. A previous study reported that peripheral β-adrenoceptor activation was involved in this phenomenon and suggested that sympathetic nervous system (SNS) nerve endings may release the neurotransmitter norepinephrine (4).

β-adrenoceptor subtypes have been identified along the gastrointestinal tracts of rats. β_1_- and β_2_-adrenergic receptors have been detected in the mucosa and musculature of the gastric antrum, and an indeterminate subtype has been detected in the muscle tissue of the pylorus (5). Additionally, elevated β_3_-adrenoceptor mRNA levels have been found in the muscle fibers of the gastric fundus and moderate levels have been found in the pylorus (6). β_3_-adrenoceptors participate in the catecholamine-induced relaxation of the gastric fundus of rats *in vitro* (7).

Many selective β-adrenergic antagonists are available, but the affinity for these receptors appears to vary among different tissues and animal species. β_1_-adrenoceptor antagonists are used for the control of cardiovascular diseases in humans, while β_2_-adrenoceptor blockade results in bronchospasm (8).

In *in vivo* rat models with altered gastric secretion or GE, the selective antagonists atenolol (β_1_-adrenergic antagonist) (9), butoxamine (β_2_-adrenergic antagonist) (10), and SR59230A (β_3_-adrenergic antagonist) (9) have been used to identify the possible β-adrenoceptor that is involved.

SNS fibers that innervate the gastrointestinal tract mostly originate from cell bodies located in the prevertebral ganglion chain (PVG). The arrangement of the rat PVG differs from that of the other mammals (11). The PVG consists of the celiac ganglion (CG, an intimately connected pair), a single superior mesenteric ganglion (SMG), a single inferior mesenteric ganglion, a pair of splanchnic ganglia in the large splanchnic nerves, and an inter-renal ganglion located in the intermesenteric nerve between the SMG and inferior mesenteric ganglion. These ganglia are organized somatotopically in such a way that the CG and SMG project fibers to the stomach, small bowel, and proximal colon, with a large part of the fibers that innervate the stomach originating in the CG (12).

In the present study, we performed the following procedures to determine which β-adrenoceptors are involved in delayed GE induced by the phenylpyrazole derivatives dipyrone, 4-aminoantipyrine, and antipyrine, and to identify participation of the prevertebral SNS in this process in rats: *i*) blockade of β-adrenoceptors with the selective antagonists atenolol, butoxamine, and SR59230A, and *ii*) concomitant surgical resection of the celiac ganglion and the superior mesenteric ganglion.

## Material and Methods

Male Wistar rats weighing 220-280 g were allowed to adapt to laboratory conditions for at least 2 weeks. The study was approved by the Ethics Committee for Animal Experimentation of the institution (protocol #1372, CEEA/UNICAMP).

The rats used in the study were maintained in individual cages. They had free access to food and water up to 24 h and 30 min before evaluation of GE between 1:00 and 4:00 pm.

Dipyrone, 4-aminoantipyrine, antipyrine, atenolol, butoxamine, SR59230A, and dimethyl sulfoxide (DMSO) were purchased from Sigma, USA. The solutions were prepared at the time of use, and when indicated, were protected from light. The doses of each drug and the method for the preparation of the solutions for intravenous (*iv)* use were established based on previous literature (9,10) and tested in the laboratory in preliminary studies.

Two experiments were carried out using β-adrenergic antagonists. The first experiment involved pretreatment with selective β_1_- and β_2_-adrenergic antagonists, and the rats were divided into the following groups (n=6): the ATE group, pretreated with atenolol (ATE) dissolved in vehicle (30 mg/kg) with a dose corresponding to 30 times the dose recommended in the literature (9); the BUT group, pretreated with butoxamine (BUT) dissolved in vehicle (25 mg/kg), corresponding to approximately three times the dose indicated in the literature (10); and the V group, pretreated with vehicle (V), which was prepared with one part DMSO + four parts sterile saline (13). In the second experiment a selective β_3_-adrenergic antagonist was tested. The rats were divided into the following groups (n=6) : the SRA group, pretreated with SR59230A (SRA) (2 mg/kg) dissolved in vehicle, corresponding to twice the dose used for rats (9); and the V group pretreated with vehicle, which was prepared as described in the first study.

High doses of selective β-adrenergic antagonists were used in these pretreatments. Preliminary assessment with these doses showed that, when care was taken to slowly administer the drugs *iv* (30-40 s) in the most distal extremity of the rat's tail, there were no systemic repercussions that might directly compromise GE, such as agitation and respiratory difficulty observed in some animals with rapid butoxamine administration. We did not include control rats pretreated with saline. The reason for this is because a preliminary study showed that addition of DMSO to vehicle at the proportion used in relation to saline (1:4) did not interfere with GE.

In the last experiment, the rats were first subjected to surgical sympathectomy. Nine days before evaluation of GE, these rats were sedated with 100 mg/kg ketamine and 10 mg/kg xylazine, which were administered intraperitoneally. These rats were divided into two groups as follows (n=10): rats were subjected to laparotomy with resection of the celiac-superior mesenteric ganglion complex (GLX) under a magnifying lens; and a sham group (SH) using a technique for surgical sympathectomy of the proximal part of the digestive tract of rats (14).

Fifteen minutes after pretreatment with the antagonists or the vehicle, the groups were treated *iv* (caudal vein) with 240 µmol/kg dipyrone (Dp), 4-aminoantipyrine (AA), antipyrine (At), or saline (S) (1 mL/kg) (4). The same treatment was applied to the GLX and SH groups. GE was determined 10 min after these treatments were administered.

GE was assessed in awake animals by determining the percentage of gastric retention (%GR) of a saline test meal that was labeled with phenol red (60 µg/mL), in a volume of 2 mL/100 g body weight, 10 min after administration. The test meal was administered by gavage using a technique that was standardized in our laboratory (15), with small modifications (16).

After this procedure, the rats were sacrificed. Those of the GLX and SH groups were examined and those with intraperitoneal adhesions were excluded. Ganglionectomy was confirmed by verifying the absence of neuronal tissue at the level of the celiac and mesenteric artery branches in the exit of the abdominal aorta (14).

Data were analyzed by ANOVA and by the Tukey's test for paired comparison (α=0.05 for both tests).

## Results

**Figure 1:**
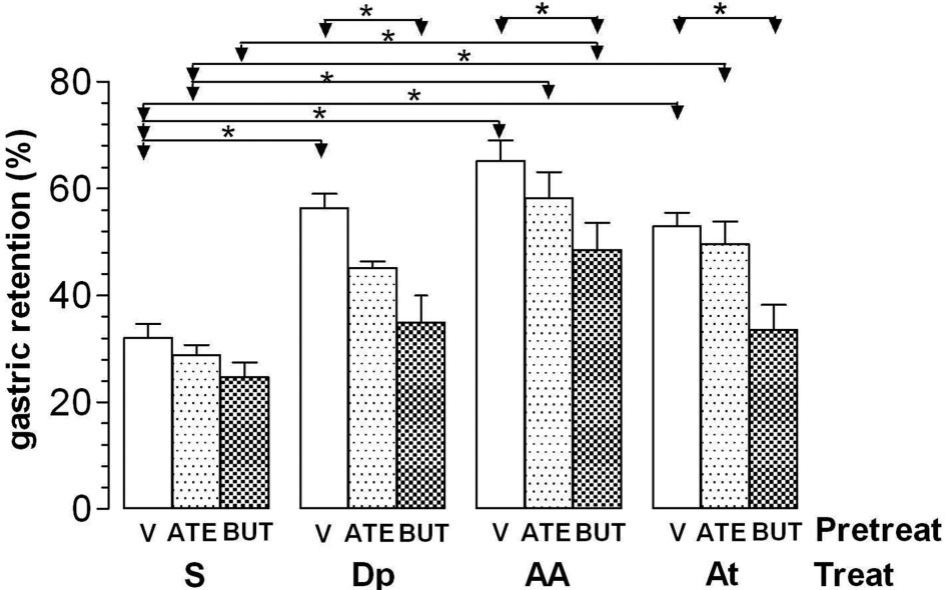
Gastric retention (%) of a saline test meal provided to rats by gavage. The rats were pretreated (Pretreat) *iv* with vehicle (V), 30 mg/kg atenolol (ATE), or 25 mg/kg butoxamine (BUT) 15 min before *iv* treatment (Treat) with saline (S), 240 µmol/kg dipyrone (Dp), 4-aminoantipyrine (AA), or antipyrine (At). The test meal was administered 10 min after treatment. Data are reported as means±SE for 6 animals per group. *P<0.05 (Tukey's test).

Figure 1 shows the results of *iv* pretreatment with ATE and BUT. Rats that were pretreated with V, ATE, or BUT and treated with S did not show significantly different %GR values (means±SE, n=6) among the groups (32.1%±2.6%, 28.8%±1.9%, and 24.7%±2.7%, respectively). Pretreatment with ATE caused no significant change in the effect of Dp (44.9%±1.3%), AA (58.2%±4.9%), or At (49.6%±4.2%) compared with controls pretreated with V and treated with the respective drugs (56.4%±2.7%, 65.2%±3.8%, and 52.9%±2.6%, respectively). The %GR in the ATE+Dp group was not significantly different from that in the ATE+S group. Pretreatment with BUT abolished the effect of Dp (35.0%±5.1%) and At (33.5%±4.7%) compared with controls pretreated with V. Pretreatment with BUT also significantly reduced (P< 0.05), but did not abolish, the effect of AA (48.0%±5.0%) compared with the V+AA control.

**Figure 2:**
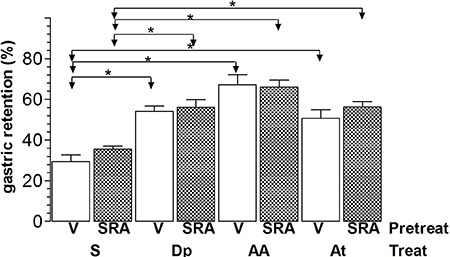
Gastric retention (%) of a saline test meal provided to rats by gavage. The rats were pretreated (Pretreat) *iv* with vehicle (V) or with 2 mg/kg SR59230A (SRA) 15 min before *iv* treatment (Treat) with saline (S), 240 µmol/kg dipyrone (Dp), 4-aminoantipyrine (AA), or antipyrine (At). The test meal was administered 10 min after treatment. Data are reported as means±SE for 6 animals per group. *P<0.05 (Tukey's test).

Intravenous pretreatment with SRA (Figure 2) did not modify %GR (means±SE, n=6) in rats that were treated with S (34.5%±3.8%) compared with controls (26.7%±5.1%). Pretreatment with SRA also did not modify the effect of Dp (56.5%±7.8%), AA (66.9%±3.3%), and At (57.2%±4%) compared with rats that were pretreated with vehicle (55.2%±1.9%, 66.8%±5%, and 54.2%±5.5%, respectively).

**Figure 3:**
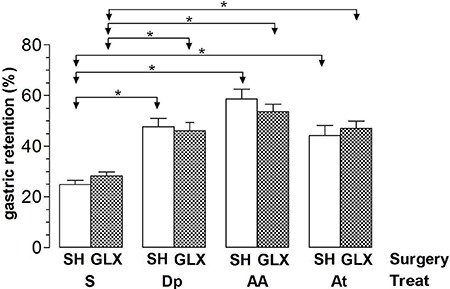
Gastric retention (%) of a saline test meal provided to rats by gavage. The rats were first subjected to surgical resection of the celiac-superior mesenteric ganglion complex (GLX) or to sham surgery (SH) and treated *iv* (Treat) with saline (S), 240 µmol/kg dipyrone (Dp), 4-aminoantipyrine (AA), or antipyrine (At). The test meal was administered 10 min after treatment. Data are reported as means±SE for 10 animals per group. *P<0.05 (Tukey's test).

Figure 3 shows the results of %GR (means±SE, n=10) in the experiment of the effect of surgical sympathectomy. Surgical resection of the celiac-superior mesenteric ganglion complex did not significantly change %GR in rats that were treated with S compared with controls (GLX+S *vs* SH+S: 28.2%±1.6% *vs* 24.9%±1.5%). Surgical resection of the celiac-superior mesenteric ganglion complex also did not change the effect of Dp (GLX+Dp *vs* SH+Dp: 46.1%±3.3% *vs* 47%±3.1%), AA (GLX+AA *vs* SH+AA: 53.5%±3% *vs* 58.6%±3.8 %), and At (GLX+At *vs* SH+At: 47%±2.9% *vs* 44.1%±4%) on GE.

## Discussion

In a previous study, *iv* pretreatment with atenolol or butoxamine at the doses of 10 and 8 mg/kg body weight, respectively, did not change the delay in liquid GE induced by dipyrone, 4-aminoantipyrine, or antipyrine in rats (Vinagre AM, personal communication). Based on this finding and evidence of participation of β-adrenoceptors in this phenomenon (4), we increased the doses of β_1_- and β_2_-adreneric antagonists in our study. Additionally, previous studies have shown variation in the selectivity and affinity of the agonists and antagonists that are available for these β-receptors (8,17).

We found that butoxamine at a dose three times higher than that used in a previous study (Vinagre AM, personal communication) blocked the delay in liquid GE induced by dipyrone and antipyrine in rats and attenuated the effect of 4-aminoantipyrine (Figure 1). This finding suggests that activation of β_2_-adrenergic receptors occurs with these drugs. These results are consistent with those observed in another study in which previous adrenergic blockade with guanethidine and pretreatment with propranolol (nonselective β-adrenergic antagonist) were studied (4).

In our study, atenolol (Figure 1) and SR59230A (Figure 2) did not significantly modify the effect of phenylpyrazole derivatives. This finding indicated unlikely activation of β_1_- or β_3_-adrenergic receptors in delayed GE, which was induced by dipyrone, 4-aminoantipyrine, or antipyrine. High doses of these antagonists might compromise these interpretations because of their potential effect on the cardiovascular system when administered alone or in combination with phenylpyrazole derivatives.

In two rat models, changes in gastrointestinal motility appear to be directly related to variation in some hemodynamic parameters. In the first model, consisting of controlled bleeding, an acute decrease in arterial pressure and central venous pressure is accompanied by increased liquid GE (18). In the second model, consisting of a reduction in arterial pressure and increased venous pressure due to an aortocaval fistula, there is a delay in GE (19). These observations suggest that distinct mechanisms may be involved in different acute hemodynamic situations, with repercussions on gastric motility.

Studies on rats investigating the effects of β-adrenergic antagonists on some cardiovascular parameters have indicated the following. *1*) acute atenolol administration (1 mg/kg by the peripheral intra-arterial route) significantly reduces cardiac output, heart rate, mean arterial pressure, and blood flow to the stomach and small bowel, increasing the total peripheral vascular resistance (20). *2*) Thirty minutes after intraperitoneal administration of 10 mg/kg butoxamine, there is no change in heart rate or mean arterial pressure compared with basal values (21). *3*) SR59230A alone, 1 mg/kg *iv*, does not modify mean arterial pressure or cardiac output in animals with liver cirrhosis (22). Therefore, among these β-adrenergic antagonists, atenolol has the greatest effect on the cardiovascular system. This suggests that, in the present study, use of atenolol at a 30 times higher dose than that demonstrating an effect on the cardiovascular system (20) may have had a greater effect on this system.

We did not find that increasing β-blocker doses alone modified liquid GE in rats. In the present study, the lack of a significant difference in %GR between control animals pretreated with vehicle or β-blockers and rats treated with saline (Figures 1 and 2) indicates that it is unlikely that these antagonists interfered *per se* with liquid GE.

Among humans, approximately 0.4% of patients who received dipyrone parenterally showed a significant reduction in systolic arterial pressure as an adverse effect (23). A single *iv* dose of 2 g dipyrone reduced the postoperative left ventricular work index by 10% in patients who had heart surgery (24). Because these changes were detected in patients with some health problems, other factors alone or in combination with this drug might be responsible for these phenomena.

In our laboratory, mean arterial pressure was measured in the left carotid artery of sedated rats (n=5-6) over 30 min after *iv* administration of vehicle (saline), 240 µmol/kg dipyrone, 4-aminoantipyrine, or antipyrine (Passafaro ACD, personal communication). Arterial pressure remained at similar levels to those measured initially (time 0) for up to 10 min after administration of these drugs in treated rats. This was followed by a discrete and gradual decrease in arterial pressure compared with controls and initial values. At 30 min after administration, the difference in arterial pressure between the groups was not significant (P<0.08, ANOVA, α=0.05). In the current study, we did not assess the hemodynamic condition of the rats. However, even if the combination of atenolol and pyrazolone derivatives substantially reduced arterial pressure, this did not interfere with the effect of treatment of the three drugs on GE (Figure 1). Therefore, there is no evidence that pretreatment with high doses of β-adrenergic antagonists, atenolol in particular, compromised our results because of their effect on the cardiovascular system.

The method of selective SNS surgery was used in our study to determine the origin of the neurotransmitter. This procedure is recommended for assessment of participation of this system in phenomena observed in a target organ (25). We simultaneously performed surgical resection of the CG and MSG, ganglia from which efferent fibers are projected towards the proximal part of the digestive tract of the rat (12,14). The results of this procedure (Figure 3) suggested that these structures were not involved in the delay in GE, which was induced by phenylpyrazole derivatives.

There are at least three other possibilities that may explain the origin of the neurotransmitter from the SNS. The first possibility is that our results do not exclude participation of the PVG in the effect of dipyrone, 4-aminoantipyrine, or antipyrine because the procedure did not involve removal of the pair of splanchnic ganglia that contain neurons, which together with those of the CG and SMG, innervate the gastrointestinal tract (12). The second possibility is that the involved neurotransmitter may originate from fibers of the sympathetic paravertebral ganglion chain for the distal part of the thorax. These fibers project towards the stomach and duodenum (12). Finally, the neurotransmitter may originate in the noradrenergic efferent fibers of the vagus nerve (26 27 28). Although there is no strong evidence of occurrence of these fibers, this possibility is supported by the fact that subdiaphragmatic vagotomy abolishes or reduces the effect of studied drugs (1 2 3). However, this possibility is not as likely if we consider that vagotomy also results in the incision of capsaicin-sensitive afferent fibers, which are involved in the effect of dipyrone, 4-aminoantipyrine, or antipyrine on GE in rats (16).
